# Impact of COVID-19 lockdown on weight loss after bariatric surgery: a retrospective single-center study with a follow-up of 3 years

**DOI:** 10.1007/s00423-025-03755-z

**Published:** 2025-08-06

**Authors:** Felix Hönes, André L. Mihaljevic, Daniel Wulff, Rami Archid, Ivan Capobianco

**Affiliations:** Department of General, Visceral and Transplantation Surgery, University Hospital Tübingen, Hoppe-Seyler-Strasse 3, 72076 Tübingen, Germany

**Keywords:** Bariatric surgery, Weight loss, COVID-19, Pandemic, Quarantine, Management

## Abstract

**Introduction:**

Obesity is a major global health concern, with bariatric surgeries commonly performed to achieve significant weight loss, reduce obesity-associated morbidity and improve quality of life. The COVID-19 pandemic disrupted daily routines, impacting physical activity and eating behaviors, potentially affecting weight outcomes. This study aims to evaluate whether the COVID-19 lockdown influenced long-term weight loss outcomes in bariatric surgery patients by comparing three-year postoperative results between patients who had surgery immediately before the lockdown and those who had surgery up to two years earlier.

**Methods:**

A retrospective cohort analysis was conducted at the University Hospital of Tuebingen. Patients were categorized into two groups: the pandemic group (surgery from October 2019 to March 2020) and the pre-pandemic group (surgery from October 2017 to March 2019). The primary outcome was weight loss, while secondary outcomes included cholesterol levels, HbA1c, and long-term complications like reflux, gallstones, and gastrointestinal bleeding. Follow-up assessments were conducted at 6 months, 1 year, 2 years, and 3 years.

**Results:**

A total of 194 patients were analyzed. Sleeve gastrectomy was performed in 72% of cases, and gastric bypasses in 28%. No significant differences were found between groups in demographics, surgical technique distribution, or follow-up rates. Weight, BMI, and excess weight loss were comparable between the two groups, with stabilization observed between 88 and 90 kg and 30–32 kg/m² from one to three years post-surgery. Complication rates and laboratory parameters (cholesterol, HbA1c, parathyroid hormone) showed no significant differences between the groups.

**Conclusion:**

The COVID-19 pandemic did not significantly affect long-term weight loss outcomes or metabolic parameters in bariatric surgery patients up to three years postoperatively. These findings suggest that despite lifestyle disruptions due to the lockdown bariatric surgery remains effective for sustained weight management.

## Introduction

Obesity is a major global health issue and increasingly important in healthcare [1]. Common surgical treatments include Sleeve Gastrectomy (SG) and Roux-en-Y Gastric Bypass (RYGB), with SG being the more frequently performed. These surgeries are generally safe, effective, and associated with long-term weight loss, low mortality, and improved quality of life and reduce obesity-associated long-term morbidity [2–6].

The COVID-19 pandemic lockdowns significantly disrupted daily routines, affecting eating behaviors and physical activity. This led to weight gain and an overall increase in obesity rates [7]. However, research on the pandemic’s impact on bariatric surgery patients is limited, with most studies focusing on outcomes within the first one to two years post-surgery. These studies generally reported no significant effect on postoperative weight loss during this early period [8–10].

Weight loss following bariatric surgery is most rapid in the first 12 months and tends to stabilize around two years. However, the period between one and three years is critical for assessing long-term weight maintenance and identifying cases of weight regain. Notably, some studies suggest that weight regain may become evident after the third year [11]. Therefore, a follow-up period extending to at least three years is essential to fully evaluate the long-term impact of the pandemic on bariatric surgery outcomes.

We aim to determine if COVID-19 pandemic affected long-term weight loss by comparing three-year outcomes between patients who underwent surgery just before the lockdown and those who had surgery up to two years earlier. In order to investigate this, all patients who underwent surgery in the six months immediately preceding the pandemic, which began in March 2020, were included.

## Materials and methods

This is a retrospective single-center cohort analysis conducted at the University Hospital of Tübingen. The study was conducted of the standards of the declaration of Helsinki and all amendments. All patients provided written consent for anonymous data collection.

We analyzed postoperative weight loss in patients who had bariatric surgery within six months before the lockdown began, namely from October 2019 to March 2020 (the pandemic group). The comparison group (the pre-pandemic group) included patients who underwent surgery during the corresponding periods in the two preceding years, specifically from October 2017 to March 2018 and from October 2018 to March 2019. Only cases during the same months were included in order to minimize seasonal influences. Revisional surgeries were excluded. There were no differences in surgical management or follow-up between the subgroups.

The primary outcome was to identify differences in weight loss between the two groups.

Secondary outcomes included differences in cholesterol levels, HbA1c, and long-term adverse events such as gallstones, reflux, dumping syndrome, gastrointestinal bleeding, and bowel obstruction. All laboratory tests were conducted at the Department of Diagnostic Laboratory Medicine, University Hospital Tuebingen, following a standardized protocol.

### Patient Follow-Up and Data Acquisition

Patients were routinely scheduled for follow-up visits at 6 months, 1 year and then annually after bariatric operation. Follow-up visits were conducted in the outpatient clinic by experienced bariatric surgeons. Examinations like endoscopy or imaging procedures, including ultrasound and CT scans, were performed only when clinically indicated. The following parameters were recorded at each follow-up appointment:

- Demographics: Age, gender, ASA (American Society of Anesthesiologists) classification,
- Date and type of surgery.
- Body weight, BMI (body mass index) and %EWL (excess weight loss).
- Laboratory Values: Cholesterol, low densitiy lipoprotein (LDL), high densitiy lipoprotein (HDL), hemoblobin A1c (HbA1c), and parathyroid hormone (PTH) levels.
- Examination findings like endoscopy or imaging by ultrasound or CT scan.
- Postoperative Complications.

Body Mass Index (BMI) is defined as the ratio of body weight in kilograms to the square of height in meters. Excess weight loss (EWL) is calculated using the formula:

%EWL = ((preoperative weight – current weight)/(preoperative weight – ideal weight)) × 100.

The ideal body weight is based on a BMI of 25 kg/m².

To further evaluate outcome trajectories over time and account for within-subject correlation, we performed linear mixed-effects models for weight, BMI, and EWL, with time, group, and their interaction as fixed effects, and subject ID as a random effect.

### Data analysis

Qualitative variables were compared using the Fisher’s exact test. Quantitative variables were evaluated using the student’s t test and, in case of non-normal distribution, the Mann-Whitney *U* test. A *p*-value of < 0.05 was considered statistically significant. All statistical computations were conducted using SPSS Statistics Software (Version 28, IBM Corporation, Armonk, NY, USA).

A post-hoc power analysis was conducted using a two-sample t-test framework to evaluate the study’s ability to detect a difference in weight loss outcomes between the pre-pandemic and pandemic groups. Based on the final sample sizes (*n* = 71 vs. 123), the study had 80% power to detect a moderate effect size of Cohen’s d = 0.42 at a two-sided alpha of 0.05. Smaller differences would likely not reach statistical significance given the available sample size.

## Results

Patient characteristics are detailed in Table 1. A total of 207 patients underwent bariatric surgery between 2017 and 2020. Thirteen patients were excluded due to previous surgeries, leaving 194 for analysis.

**Table 1:** Demographic data

		Overall Mean (SD) *n* = 194	Pandemic Group Mean (SD) *n* = 71	Pre-Pandemic Group Mean (SD) *n* = 123	*P* value
Number per quarter:					
- Pre-Pandemic:	**Q4 2017**	23 (12)		23 (19)	
	**Q1 2018**	29 (15)		29 (24)	
	**Q4 2018**	37 (19)		37 (30)	
	**Q1 2019**	34 (18)		34 (28)	
- Pandemic:	**Q4 2019**	49 (25)	49 (69)		
	**Q1 2020**	22 (11)	22 (11)		
Gender					1.0^&^
- Female		133 (69)	49 (69)	84 (68)	1.0^&^
- Male		61 (31)	22 (31)	39 (32)	1.0^&^
Age (y, median)		42.0 (18.0)	42.1 (12.6)	42.0 (18.0)	1.0^‡^
Procedure:					0.835^&^
- Sleeve-gastrectomy		139 (72)	52 (75)	88 (72)	
- Roux-Y-Gastric bypass		55 (28)	19 (27)	36 (28)	
Simultanous procedures:		7 (3.6)	7 (9.9)	0 (0)	1:0**
- Cholecystectomy		1 (12)	1 (12)	0 (0)	0.366**
- Hiatoplasty		6 (75)	6 (75)	0 (0)	0.002**
- IPOM		1 (12)	1 (12)	0 (0)	0.366**
ASA classification					0.758^&^
− 2		108 (56)	38 (54)	70 (56)	0.871**
− 3		86 (44)	33 (46)	53 (43)	0.655**
Follow-up					
- ½ y		145 (74.7%)	57 (80.3%)	88 (71.5%)	0.230**
− 1 y		162 (83.5%)	60 (84.5%)	102 (83.0%)	0.843**
− 2 y		117 (60.3%)	46 (64.8%)	71 (57.8%)	0.363**
− 3 y		99 (51.0%)	36 (50.7%)	63 (51.2%)	0.979**

Table 1 describes the demographic data of the patients. The number of pandemic and pre-pandemic groups and patients per quarter, gender, age, surgical technique, total surgical ASA classification and the proportion of follow-up up to a total of 3 years postoperatively are listed. In addition to the absolute number, the standard deviations (SD) are given. A *p*-value of > 0.05 is considered as significant difference

Most patients were female (69%), with no significant difference in sex distribution between groups. There were also no significant differences in age (42.1 vs. 42.0 y *p* = 1.0) or ASA classification (*p* = 0,871 for ASA 2, *p* = 0,0655 for ASA 3). Simultaneously performed operations were performed only in the pre-pandemic group (1 cholecystectomy, *p* = 0,366, 6 hiatoplasty, *p* = 0,002, 1 Hernia, *p* = 0,366).

55 patients had gastric bypass (28%), and 139 had sleeve gastrectomy (72%).

Follow-up rates were 83.5% at one year, 60% at two years, and 51% at three years after surgery. No significant differences were found in the distribution of surgical techniques, ASA classification, or follow-up rates (Table 1).

The overall complication rate was 11.9%. Table 2 shows short-term complications including hematoma (*n* = 1, 0.5%) and gastrointestinal bleeding (*n* = 1, 0.5%) and long-term complications involved reflux disease (*n* = 8, 4.1%), gastritis (*n* = 2, 1.0%), abdominal wall hernia (*n* = 5, 2.6%), and gallbladder or bile duct disorders, such as choledocholithiasis, which required cholecystectomy (*n* = 6, 3.1%). The overall complication rate did not differ between the pre-pandemic group and the pandemic group (Table 2).

**Table 2:** Postoperative outcomes and interventions

Postoperative outcomes	(*n*, %) Overall *n* = 194	Pandemic Group (*n*, %) Overall *n* = 71	Pre-Pandemic Group (*n*, %) Overall *n* = 123	*P* value
Postoperative complications	23 (11.9)	10 (14.1)	13 (10,6)	0.495**
• Choledocho-/Cholecystolithiasis	6 (3.1)	1 (1.4)	5 (4.1)	0.179**
• GI-bleeding	1 (0.5)	0 (0.0)	1 (0.8)	0.22**
• Gastritis	2 (1.0)	0 (0.0)	2 (1.6)	0.497**
• Hematoma	1 (0.5)	1 (1.4)	0 (0.0)	0.434**
• Hernia	5 (2.6)	4 (5.6)	1 (0.8)	0.151**
• Reflux disease	8 (4.1)	4 (5.6)	4 (3.3)	0.712**

Table 2 shows the peri- and postoperative complications of all patients and divided into the pre-pandemic and pandemic group. The percentage within the respective group (all patients, pandemic group, pre-pandemic group) is shown in brackets. ** Fisher’s Exact Test

Choledocholithiasis (1.4% vs. 4.1%, *p* = 0.179), and reflux disease (*n* = 4 vs. 4, *n* = 0,0712) were the most common, particularly in the pre-pandemic group, while hernias (5.6% vs. 0,8%, *p* = 0.151) and reflux disease (5.6% s. 3.3%, *p* = 0.712) were more frequent in the pandemic group.

Preoperative weight (137.4 vs. 136.0, *p* = 1,0) or BMI (48.7 vs. 49.0, *p* = 0,584) did not differ between the groups. No significant differences in postoperative weight, BMI, or excess weight loss were observed between the two groups, preoperatively and at each postoperative follow-up time point. Weight and BMI stabilize between 88 and 90 kg (*p* = 1.0) and 30–32 kg/m² (*p* = 1.0), respectively, from one to three years post-surgery. Excess weight loss remains between 68% and 72% in both groups without significant differences (68.9% vs. 68.2%, *p* = 0.689) (Table 3).

**Table 3:** Patient follow-up

Parameter	Time point	Overall Mean (SD) *n* = 194	Pandemic Group Mean (SD) *n* = 71	Pre-Pandemic Group Mean (SD) *n* = 123	*P* value
Weight (kg)	Preoperative	135.0 (33.8)	137.4 (22.7)	136.0 (35.5)	1.0^‡^
	½ y	99.0 (27.0)	100.0 (17.6)	100.0 (30.0)	1.0^‡^
	1 y	89.0 (25.0)	90.3 (17.0)	90.5 (24.5)	1.0^‡^
	2 y	88.0 (26.0)	90.6 (18.9)	89.0 (24.0)	1.0^‡^
	3 y	90.0 (23.5)	89.7 (15.4)	90.0 (24.0)	1.0^‡^
BMI (kg/m ^2^ )	Preoperative	48.7 (7.2)	48.4 (6.4)	49.0 (7.7)	0.584^§^
	½ y	35.8 (5.7)	35.2 (5.7)	36.2 (6.0)	0.41^§^
	1 y	31.9 (5.9)	31.3 (5.2)	32.8 (5.8)	1.0^‡^
	2 y	31.0 (7.0)	31.8 (5.7)	30.8 (5.7)	1.0^‡^
	3 y	31.8 (5.2)	31.6 (4.9)	32.0 (5.4)	0.737^§^
EWL (%)	½ y	56.0 (18.0)	56.0 (15.0)	55.5 (18.2)	1.0^‡^
	1 y	69.0 (26.0)	71.6 (20.3)	68.0 (25.0)	1.0^‡^
	2 y	71.6 (22.7)	70.1 (25.2)	72.6 (20.9)	0.568^§^
	3 y	68.9 (22.9)	70.1 (22.4)	68.2 (23.2)	0.689^§^
Cholesterol (mg/dl)	Preoperative	174.0 (37.6)	161.8 (35.4)	180.9 (37.1)	0.001^§^
	½ y	170.1 (35.8)	168.0 (35.9)	171.2 (35.9)	0.592^§^
	1 y	177.3 (35.8)	173.1 (33.3)	179.7 (37.2)	0.264^§^
	2 y	180.3 (39.8)	172.1 (36.4)	185.5 (41.3)	0.079^§^
	3 y	179.0 (42.2)	175.0 (37.4)	181.0 (43.0)	1.0^‡^
HDL (mg/dl)	Preoperative	41.0 (11.0)	38.4 (8.2)	43.0 (12.0)	1.0^‡^
	½ y	48.6 (10.5)	49.5 (11.3)	48.1 (10.1)	0.426^§^
	1 y	54.5 (15.0)	54.5 (12.2)	54.5 (17.0)	1.0^‡^
	2 y	58.0 (17.0)	57.6 (14.4)	58.5 (14.5)	1.0^‡^
	3 y	62.3 (14.9)	62.1 (15.4)	62.5 (14.7)	0.897^§^
LDL (mg/dl)	Preoperative	117.3 (35.5)	111.6 (34.1)	120.5 (35.9)	0.096^§^
	½ y	110.2 (35.0)	110.6 (35.1)	110.0 (35.1)	0.914^§^
	1 y	113.0 (35.7)	110.9 (32.9)	114.3 (37.3)	0.557^§^
	2 y	113.8 (38.3)	108.3 (34.5)	117.4 (40.5)	0.213^§^
	3 y	116.0 (36.5)	110.7 (36.5)	119.2 (36.5)	0.269^§^
Triglyceride (mg/dl)	Preoperative	116.0 (70.0)	105.5 (65.5)	126.0 (73.0)	1.0^‡^
	½ y	93.5 (45.5)	89.0 (42.0)	98.0 (49.0)	1.0^‡^
	1 y	86.0 (48.2)	90.7 (30.7)	88.5 (49.2)	1.0^‡^
	2 y	83.5 (45.5)	92.7 (38.0)	86.0 (44.5)	1.0^‡^
	3 y	88.0 (57.0)	103.7 (47.0)	82.0 (60.0)	1.0^‡^
HbA1c (%)	Preoperative	5.8 (1.0)	5.9 (0.9)	5.7 (1.0)	1.0^‡^
	½ y	5.4 (0.5)	5.4 (0.5)	5.4 (0.5)	1.0^‡^
	1 y	5.4 (0.5)	5.3 (0.5)	5.4 (0.5)	1.0^‡^
	2 y	5.4 (0.5)	5.4 (0.5)	5.4 (0.5)	1.0^‡^
	3 y	5.5 (0.5)	5.5 (0.3)	5.5 (0.5)	1.0^‡^
Parathormone (pmol/l)	Preoperative	5.8 (3.1)	5.7 (2.3)	6.0 (3.5)	1.0^‡^
	½ y	5.5 (2.6)	5.6 (2.4)	5.3 (2.7)	1.0^‡^
	1 y	5.2 (2.8)	5.2 (2.7)	5.4 (2.8)	1.0^‡^
	2 y	5.8 (2.9)	6.0 (2.4)	5.8 (3.1)	1.0^‡^
	3 y	5.9 (3.9)	5.8 (3.0)	6.2 (5.1)	1.0^‡^

Table 3 shows the mean values for weight, BMI, excess weight loss (EWL), cholesterol, HDL, LDL, triglycerides, HbA1c and parathyroid hormones for all patients and for the pre-pandemic group and the pandemic group. The standard deviations (SD) are in brackets. A *p*-value of < 0,05 was considered significant. ** Fishers’s Exact-Test; ^‡^ Mann-Whitney-U;^;§^t-test

Cholesterol, LDL, HDL, and triglycerides were measured at each follow-up visit. Similar to weight loss, no significant differences were found between the groups in these lipid levels in the postoperative time.

HbA1c, reflecting long-term average blood glucose levels, decreased in both groups shortly after surgery, with no significant differences between them.

Parathyroid hormone levels, an indicator of blood calcium, showed no differences between groups and remained stable throughout the study period.

Baseline characteristics between the pre-pandemic group (*n* = 123) and pandemic group (*n* = 71) were well-matched with no significant differences observed (Table 1). In addition, we compared baseline characteristics and early postoperative weights between patients who completed the 3-year follow-up and those who were lost to follow-up. A significant difference was observed in preoperative BMI (*p* = 0.014), BMI at 1/2 years (*p* = 0.004), and LDL levels (preoperative *p* = 0.045, 1-year *p* = 0.043). These differences suggest that patients lost to follow-up may have had distinct baseline characteristics compared to completers. As such, the potential impact of missing data on study outcomes is acknowledged, and these baseline differences are considered in the interpretation of the results.

Mixed-effects models confirmed that there were no statistically significant differences in the trajectory of weight, BMI, or EWL over time between the pre-pandemic and pandemic groups (Table 4).

**Table 4:** Mixed-effects model

Outcome	Intercept (β₀)	Time (months, β₁)	Group (pre-pandemic, β₂)	Time × Group (β₃)	*p*-value for Interaction	Conclusion
Weight	68.82 (*p* = 0.002)	−0.57 (*p* = 0.61)	5.45 (*p* = 0.86)	−0.07 (*p* = 0.97)	0.97	NS
BMI	23.60 (*p* = 0.006)	−0.19 (*p* = 0.65)	0.89 (*p* = 0.94)	−0.02 (*p* = 0.97)	0.97	NS
EWL	38.19 (*p* < 0.001)	0.29 (*p* = 0.63)	1.42 (*p* = 0.93)	−0.07 (*p* = 0.95)	0.95	NS
Chol	99.97 (*p* = 0.009)	0.18 (*p* = 0.922)	6.47 (*p* = 0.905)	−0.002 (*p* = 1.000)	1.000	NS
HDL	26.43 (*p* = 0.018)	0.38 (*p* = 0.492)	2.26 (*p* = 0.887)	−0.07 (*p* = 0.926)	0.926	NS
LDL	72.40 (*p* = 0.001)	0.01 (*p* = 0.994)	3.11 (*p* = 0.919)	0.07 (*p* = 0.965)	0.965	NS
TAG	72.26 (*p* < 0.001)	−0.11 (*p* = 0.870)	12.88 (*p* = 0.518)	−0.50 (*p* = 0.614)	0.614	NS
HbA1c	3.17 (*p* = 0.020)	−0.01 (*p* = 0.889)	−0.03 (*p* = 0.988)	0.00 (*p* = 0.974)	0.974	NS
PTH	3.92 (*p* < 0.001)	0.01 (*p* = 0.758)	0.19 (*p* = 0.863)	0.02 (*p* = 0.739)	0.739	NS

Table 4 shows a mixed-effects model estimating for each outcome variable assessing changes over time and between groups (pre-pandemic vs. pandemic). The table reports fixed-effect coefficients for the intercept (baseline value), time (months), group (pre-pandemic), and the interaction between time and group, along with corresponding *p*-values. “NS” denotes “Not Significant,” indicating a *p*-value greater than 0.05 for the interaction term

The observed difference in weight loss between groups was minimal, with a standardized effect size of Cohen’s d = −0.085, corresponding to a very small, non-significant difference. The post-hoc power to detect this observed effect was only 8.8%, indicating that such small differences would not be reliably detected. However, the study was sufficiently powered (80%) to detect moderate group differences (Cohen’s d ≥ 0.42), suggesting that any true effect of the COVID-19 pandemic on postoperative weight loss, if present, is likely to be small and clinically negligible.

## Discussion

It is well-documented that changes in daily habits, such as those occurring during holidays, could be linked to weight gain [12]. Similarly, many people experienced weight increases during the pandemic, notably in spring 2020 [13]. The COVID-19 pandemic has been associated with reduced physical activity, poor eating habits, mood disturbances, and anxiety, all of which can lead to weight loss or lead to weight regain [14].

Some studies have reported reduced weight loss during the pandemic following bariatric surgery [15, 16]. To our knowledge there are only data published describing a period up to two years after the start of the pandemic [8–10]. Here we describe longer term data following up to 3 years postoperatively.

There are various definitions for pathological weight regain after bariatric surgery [17], making it challenging to establish a uniform criterion. Studies consistently show that maximal weight loss typically occurs between 1- and 3-years post-surgery. Weight loss tends to slow down in the first year, with the lowest point generally around 1-year post-surgery. After 3 years, body weight often begins to increase. Different studies have proposed various time points for weight loss failure up to 3 years after surgery [2, 11, 18]. Thus, a follow-up period of just one to two years may not be sufficient to detect weight loss failure. However, research with longer follow-up periods has found no significant differences in weight loss after one or two years [8–10].

**Fig. 1:**
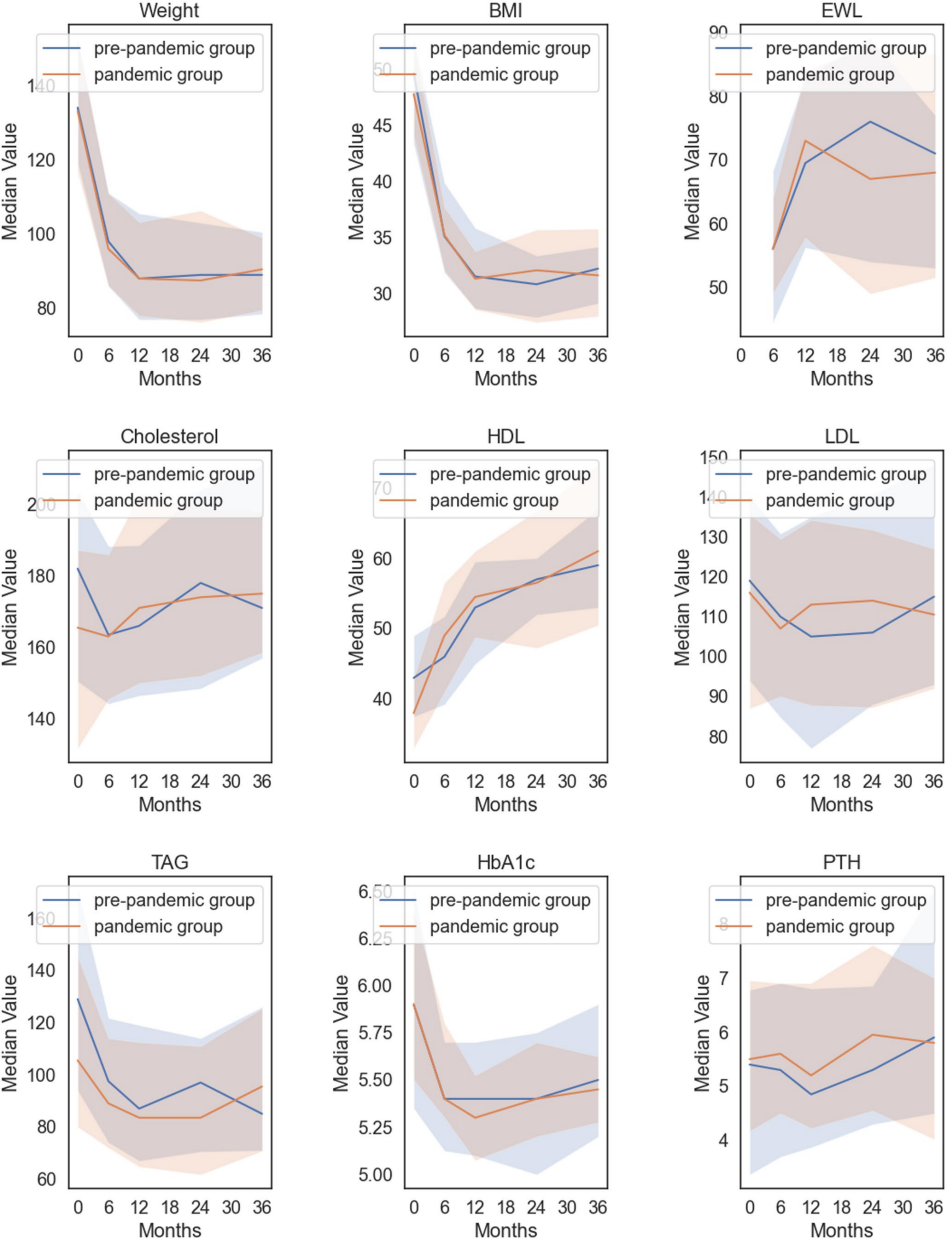
Median values over time for Weight, BMI, EWL, Cholesterol, HDL, LDL, Triglycerides, HbA1c, and Parathormone. The blue line represents the pre-pandemic cohort, while the orange line represents the pandemic cohort. Shaded areas indicate the interquartile range (IQR, 25 th–75 th percentile), illustrating variability in the distributions at each time point

This study is among the first to track weight progression over a full three years. Similar to studies by Huang, Pereira, and Moreno et al., we found no significant differences in weight trends between patients who had surgery immediately before the pandemic and those who had it earlier [8–10]. However, a longer follow-up is crucial to assess whether significant weight loss failure has occurred. Since all patients were ultimately exposed to pandemic conditions by the end of the three-year follow-up, our comparison focuses on the impact of the pandemic during the early postoperative period. To minimize seasonal effects, cases were matched by calendar months. Additionally, outcomes across the pre-pandemic subgroups were consistent. Therefore, it seems that the pandemic during the early postoperative phase after bariatric surgery did not significantly impact weight loss outcomes (Fig. 1).

Given the comparability of baseline characteristics such as age, gender, type of surgery, and preoperative weight, the pandemic represents the primary differentiating factor between the two groups (Table 1). Despite the follow-up rate declining from approximately 83.5% one-year post-surgery to 51% three years after surgery (Table 1), our data remain meaningful, as similar follow-up rates are commonly reported in other studies. A comparison of baseline characteristics revealed significant differences between patients who completed the 3-year follow-up and those lost to follow-up. Specifically, preoperative BMI and LDL and BMI at 6 months and LDL at one year after surgery were significantly different between the two groups. This suggests that the non-completers may have had higher BMI and LDL levels at baseline, potentially influencing their likelihood of completing the follow-up. This finding can affect the progression slightly both subgroups in the same way. Despite everything, true long-term weight outcomes could probably differ however, which unfortunately can not be answered due to the retrospective design of the study.

During the three-year follow-up period, weight loss is often influenced by entero-neurohumoral mechanisms [19]. Immediate weight regain post-surgery is not strongly prevented by life interventions [20]. Only sometime after weight-reducing operation maladaptive eating behaviors or lack of physical activity can lead to weight regain [21]. Huang et al. reported a significant impact of the COVID-19 pandemic on bariatric patients’ behaviors. Over half of the patients experienced a decrease in physical activity, and more than a third reported worsening eating habits. Despite these changes, weight loss one year after surgery was similar to that of patients operated on before the pandemic began [8]. Our study confirms that this similarity extends also to the first three years post-surgery, as shown in Table 3. We found no significant differences in weight, BMI, or excess weight loss up to three years after surgery.

Several other factors influence weight loss after bariatric surgery, including anatomical and surgical aspects such as gastric dilation, demographic factors like age, sex, and socio-economic status, preoperative BMI, comorbidities, patient behavior, adherence to dietary guidelines, social support, physical activity, and hormonal status [11]. In our cohort, there were no significant differences in age, gender distribution, type of operation, preoperative BMI, or preoperative blood lipid levels, as shown in Tables 1 and 3 as well as in Fig. 1. We therefore assume these factors are evenly distributed across the groups. Given the imprecision of retrospectively assessing these factors, they were not included in further analysis.

Successful weight loss partially relies on physical activity. El Ansari et al. review supports this but highlights limitations in data validity, such as discrepancies between self-reported and measured physical activity and variability in activity duration and intensity. Most patients are insufficiently active, with only a small fraction meeting health promotion guidelines of 150 min per week, even without the pandemic’s influence [22, 23]. Additionally, up to 20% of patients experience suboptimal weight loss after bariatric surgery, regardless of their physical activity levels [24]. A retrospective assessment of physical activity could not be objectively performed. Nevertheless, our results suggest that the bariatric procedure itself plays a predominant role in weight progression and appears resilient even under lifestyle restrictions imposed during the pandemic. The influence of factors such as support groups, media, or patients’ motivation to mitigate lockdown effects remains unclear. Future studies should further investigate weight loss and potential regain over longer postoperative periods.

In our study, we examined the impact of the pandemic on blood values related to dyslipidemia, diabetes, and parathyroid hormone levels as a marker for calcium deficiency. The data are illustrated in Table 3 and the course of values over time in Fig. 1 showing similar progressions in both of the groups. A case-control study of 102 patients found that the COVID-19 lockdown negatively affected weight loss in the first year after sleeve gastrectomy but showed no differences in remission rates for type 2 diabetes, hypertension, or dyslipidemia [16]. Moreno et al. also assessed remission rates for high blood pressure, diabetes, and dyslipidemia, finding similar rates under pandemic and non-pandemic conditions. Similarly, our study detected no significant differences in dyslipidemia progression, long-term glucose levels, or parathyroid hormone levels between the groups.

No direct comparison was made between gastric bypass and sleeve gastrectomy due to small group sizes, particularly for gastric bypass, which limited meaningful statistical analysis. Moreno et al. found no differences in weight loss between sleeve gastrectomy and gastric bypass during the pandemic [9], consistent with other studies showing minimal short-term differences between these procedures [25].

This study has several limitations: it is single-center, involves small sample sizes, and has low follow-up rates, although these are comparable to other studies. We did not differentiate between the surgical techniques or between the quarter immediately before the pandemic began (Q1 2020) and the previous quarter (Q4 2019). The group sizes for each specific category were too small to permit a meaningful analysis. Therefore, this study cannot address potential differences between surgical techniques. Likewise, comparisons between the mentioned quarters were not feasible. However, such differences seem unlikely, given that no significant variation in weight progression was observed at any time point. The impact of medication on blood lipids, blood glucose, and weight progression could not be retrospectively assessed, a challenge also noted in other studies due to the complex interactions of multiple medications. Moreover, this was only a secondary endpoint and was not the focus of the study. A major bias of the study is the lack of a survey of physical activity, which could only have been carried out with insufficient accuracy retrospectively.

## Conclusion

In line with other research, our study suggests that the COVID-19 pandemic did not significantly affect weight loss in the first three years after bariatric surgery. This finding is limited to the influence of the pandemic in the early phase after bariatric surgery. Although not all influencing factors could be taken into account, this study indicates the high potency on weight progression of bariatric surgery, which exceeds even the impact of the pandemic. Therefore, we conclude that successful weight loss was achieved by bariatric surgery even under pandemic conditions in the early phase after bariatric surgery.

## Data Availability

No datasets were generated or analysed during the current study.
